# Possibility of brigatinib‐based therapy, or chemotherapy plus anti‐angiogenic treatment after resistance of osimertinib harboring *EGFR* T790M‐*cis*‐C797S mutations in lung adenocarcinoma patients

**DOI:** 10.1002/cam4.4336

**Published:** 2021-10-06

**Authors:** Yaning Yang, Haiyan Xu, Li Ma, Lu Yang, Guangjian Yang, Shuyang Zhang, Xin Ai, Shucai Zhang, Yan Wang

**Affiliations:** ^1^ Department of Medical Oncology National Cancer Center/National Clinical Research Center for Cancer/Cancer Hospital Chinese Academy of Medical Sciences and Peking Union Medical College Beijing China; ^2^ Department of Comprehensive Oncology National Cancer Center/National Clinical Research Center for Cancer/Cancer Hospital Chinese Academy of Medical Sciences and Peking Union Medical College Beijing China; ^3^ Department of Medical Oncology Beijing Tuberculosis and Thoracic Tumor Research Institute Beijing Chest Hospital Capital Medical University Beijing China

**Keywords:** *cis*‐C797S, *EGFR*, non‐small cell lung cancer, resistance, treatment

## Abstract

**Background:**

There was no standard treatment for patients who acquired resistance to osimertinib mediated by epidermal growth factor receptor (*EGFR*) T790M‐*cis*‐C797S. The aim of this study was to investigate the association between different therapeutic strategies and survival outcomes among these patients.

**Methods:**

This retrospective cohort study analyzed 46 patients with metastatic lung adenocarcinoma and *EGFR* T790M‐*cis*‐C797S after osimertinib progression from January 1, 2017 to October 31, 2020. Among them, 13 patients received brigatinib‐based therapy, 23 patients received chemotherapy in combination of anti‐angiogenics or not, and 10 patients received other targeted treatments like dacomtinib, bevacizumab, or a combined therapy of osimertinib and other targeted drugs.

**Results:**

Compared to other targeted therapy, brigatinib‐based therapy (median progression‐free survival [mPFS]: 4.40 vs. 1.63 months, hazard ratio [HR] = 0.39, 95% confidence interval [CI]: 0.21–0.73, *p* = 0.001) and chemotherapy‐based treatment (mPFS: 4.70 vs. 1.63 months, HR = 0.18, 95% CI: 0.06–0.50, *p* < 0.001) presented a better survival outcome and there was no significant difference between brigatinib‐based therapy and chemotherapy‐based treatment (mPFS: 4.40 vs. 4.70 months, HR = 1.24, 95% CI: 0.57–2.67, *p* = 0.58). Chemotherapy combined with anti‐angiogenics achieved a better efficacy than only chemotherapy (mPFS: 5.50 vs. 1.03 months, HR = 0.30, 95% CI: 0.11–0.83, *p* = 0.02). Patients carrying *EGFR* exon 19 deletion mutation had a longer PFS than those who harboring *EGFR* exon 21 p.L858R mutation (4.57 vs. 1.03 months, HR = 0.18, 95% CI: 0.06–0.54, *p* = 0.001), no matter they received brigatinib‐based therapy (mPFS: 5.00 vs. 3.23 months, HR = 0.19, 95% CI: 0.01–0.96, *p* = 0.05) or chemotherapy‐based treatment (mPFS: 7.23 vs. 1.03 months, HR = 0.05, 95% CI 0.01–0.49, *p* < 0.001).

**Conclusion:**

Brigatinib‐based therapy and chemotherapy plus anti‐angiogenics could be considered beyond progression from osimertinib therapy. For patients harboring *EGFR* exon 19 deletion/T790M/*cis*‐C797S mutation, the clinical efficacy was superior to patients harboring *EGFR* exon 21 p.L858R/T790M/*cis*‐C797S mutation.

## Graphical abstract

Epidermal growth factor receptor (*EGFR*) C797S mutation plays an important role on the resistance of osimertinib in advanced non‐small‐cell lung cancer patients. The clinical efficacy of various treatments was evaluated in 46 patients carrying *EGFR* T790M‐*cis*‐C797S mutation. We demonstrated the possibility of brigatinib‐based therapy and chemotherapy plus anti‐angiogenic might bring better clinical outcomes in those patients and the co‐occurring *EGFR* 19del might have a better survival than *EGFR* 21L858R.

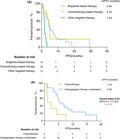

## 1 INTRODUCTION

In the Chinese population, non‐small cell lung cancer (NSCLC) harboring an epidermal growth factor receptor (*EGFR*)‐activating mutation accounts for 40% to 65%.^1^ Despite potent efficacy with first‐line *EGFR* tyrosine kinase inhibitors (*EGFR*‐TKIs), a majority of patients eventually developed resistance after around 1 year.^2^, ^3^, ^4^ Approximately 60% of patients are found to have a *EGFR* exon 20 p.T790M (*EGFR* T790M) mutation in the gene coding *EGFR* at the time of progression and osimertinib was substantially effective to target T790M.^5^ However, it is inevitable to encounter resistance generated by various mechanisms and the most common is the emergence of *EGFR* C797S mutation.^6^, ^7^ Depending on the allelic relationship with T790M, C797S is defined as *cis*‐C797S or *trans*‐C797S^8^ which have clinical implications that T790M‐*trans*‐C797S maintains sensitivity to the combination of first‐ and third‐generation of *EGFR* TKIs, whereas T790M‐*cis*‐C797S mediate resistance to osimertinib and non‐response to first, second, or third generation of *EGFR*‐TKIs. Up to now, there is not a standard therapy guideline for patients harboring *EGFR* T790M‐*cis*‐C797S and as a result of lack of appropriate targeted therapy, the most common treatment is chemotherapy. However, the treatment efficacy was not clear yet.

To substantially promote survival outcomes of these patients, we urgently need to investigate novel therapeutic strategies. Brigatinib (AP26113) was a TKI targeted either anaplastic lymphoma kinase (*ALK*) or *EGFR* and cetuximab, an antibody that blocks *EGFR* dimerization, hampering the kinase uniformly susceptible to the allosteric agent, both are reported to overcome resistance in preclinical research.^9^, ^10^ Moreover, several clinical studies have observed benefits from the treatment of brigatinib/cetuximab regimen.^11^, ^12^ Furthermore, in a retrospective research, five patients who received brigatinb combination with cetuximab achieved a favorable outcome that progression‐free survival (PFS) reached 14 months and 60% of objective response rate.^13^

In conclusion, previous studies have introduced potential clinical response strategies for patients harboring *EGFR* T790M‐*cis*‐C797S mutation. Albeit, the existing studies were small‐sample‐sized and needed more powerful validation to identify clinical efficacy in these patients. Our study aimed to illustrate the landscape of various therapeutic strategies in a real‐world setting. Then we dedicated to comparing their efficacy to bring reference evidence for clinical practice.

## 2 PATIENTS AND METHODS

### 2.1 Patients’ selection and data collection

This study retrospectively reviewed a total of 46 postoperative recurrent or metastatic adenocarcinoma patients who carried *cis*‐C797S mutation when progressed beyond osimertinib treatment. All the patients received treatment in National Cancer Center/National Clinical Research Center for Cancer/Cancer Hospital, Chinese Academy of Medical Sciences and Peking Union Medical College and Beijing Chest Hospital, Capital Medical University from January 1, 2017 to October 31, 2020 when the presence of C797S mutation. The presence of measurable lesions according to the Response Evaluation Criteria in Solid Tumors (RECIST 1.1). Next‐generation sequencing was used to detect *EGFR cis*‐C797S mutation. Patients with brain metastases or meningeal metastases were eligible at baseline. The following chart of this study was illustrated in Table S1.

### 2.2 Treatment assessment and definitions

Depending on the treatment, we divided patients into three groups: brigatinib‐based therapy, chemotherapy‐based treatment combined with anti‐angiogenics or not, and other targeted therapies like dacomtinib, bevacizumab, or a combined therapy of osimertinib and other targeted drugs. Patients in the brigatinib‐based therapy group received brigatinib orally once daily at an initial dose of 90 mg for 7 days and increased to 180 mg from day 8 onward if tolerated and when cetuximab used at a dose of 500 mg/m^2^, administrated intravenously on days 1 and 8 of a 21‐day cycle. Patients received chemotherapy and targeted therapy at a standard dose according to the China Society of Clinical Oncology guideline or National Comprehensive Cancer Network (NCCN) guideline. Imaging examination at baseline was confirmed with measurable target lesions documented by computed tomography images of the chest and abdomen, brain magnetic resonance imaging, and whole‐bone scans. Whether the response presented a complete response (CR), partial response (PR), stable disease (SD) or progressive disease has been evaluated every 2 months according to the RECIST version 1.1.

The primary endpoint was PFS which was defined as the duration from the initiation of therapy to the date of disease progression or death. Overall survival (OS) was the period from the date of treatment to death or last follow‐up that August 1st, 2021. Disease control rate (DCR) was defined as the percentage of CR, PR, and SD. Smokers were defined as current or former smokers and non‐smokers were the people who smoke less than 100 cigarettes in their lifetime. All clinical data were collected from electronic records. As an observational study, this research was exempted from obtaining patients’ informed consent without therapeutic intervention, and this research was approved by the Research Ethics Board of Cancer Hospital and conducted in accordance with the Declaration of Helsinki as well.

### 2.3 Tissue processing and organoid culture

Lung cancer organoids were derived from pleural effusion of one lung cancer patient who carried *EGFR* T790M‐*cis*‐C797S mutation at the National Cancer Center/National Clinical Research Center for Cancer/Cancer Hospital, Chinese Academy of Medical Sciences and Peking Union Medical College, Beijing, China. On arrival, tumor tissues were washed with cold phosphate‐buffered saline, cut into small pieces, washed with Advanced DMEM/F12 (Thermo Fisher Scientific; containing 1× Glutamax, 10 mM HEPES and antibiotics) and digested with collagenase (Sigma‐Aldrich; Cat. No. C9407, 2 mg/ml) for 1–2 h at 37°C. After washing twice with fresh medium (2% fetal calf serum) and centrifugation (17.8 *g*, 4 min), dissociated cells were seeded into growth factor‐reduced matrigel (Corning, Inc.) with the presence of Advanced DMEM/F12 at 37°C for 30 min. Next, the surface of a solidified mixture of cell suspension/Matrigel was sealed with complete human organoid medium (500 μl), which was comprised of advanced DMEM/F12 supplemented with series additives as described by Lampis et al.,^14^ with the replacement of every 3 days. When the organoids ranged up to 200–500 μm in diameter (about 1 week), organoids were dissociated and passaged weekly using TrypLE Express (Gibco). The patient‐derived tumor organoids (PDTO, 2 × 10^6^ cells/tube, p3) were conducted using the Recovery Cell Culture Freezing Medium (Gibco) and stored at −80°C before a drug screening.

### 2.4 Statistical analysis

SPSS version 23.0 (SPSS, Inc.) was used for statistical analysis. Univariate analysis was performed using the log‐rank test, and multi‐variants analysis using a Cox proportional hazard regression model. PFS was estimated using Kaplan–Meier analysis, and the log‐rank test was utilized to compare the differences in survival distributions between groups. All statistical tests with two‐sided *p* < 0.05 were considered statistically significant. Variables included age, gender, smoking history, Eastern Cooperative Oncology Group (ECOG) scores, metastatic sites, mutation landscape, and treatment regimen. Graphpad 5.0 was used to present survival curves.

## 3 RESULTS

### 3.1 Baseline characteristics

**FIGURE 1:**
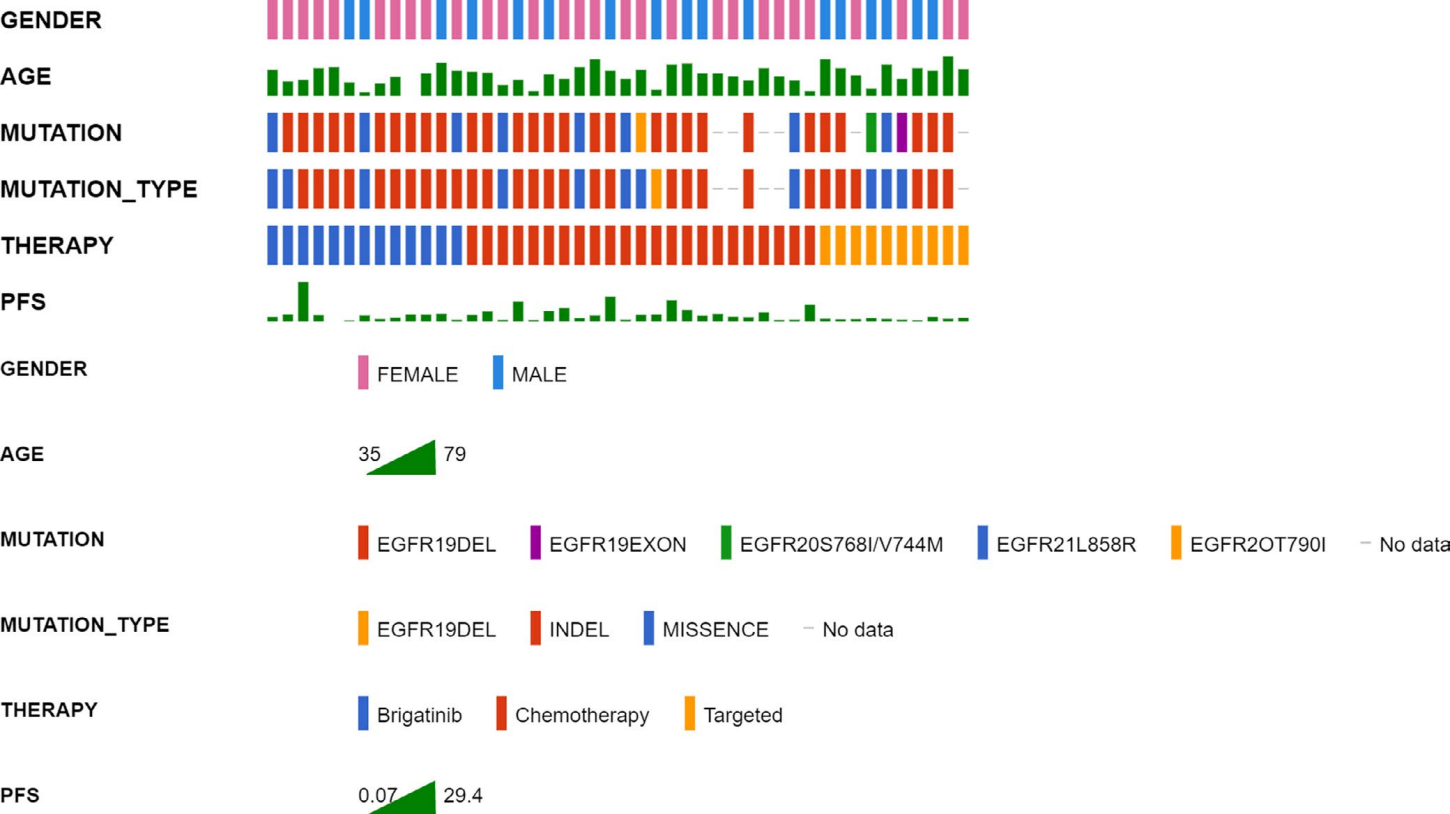
Baseline characteristics of all patients

This study collected a total of 46 patients harboring *EGFR* T790M‐*cis*‐C797S mutation, including 17 male (37.0%) and 29 female (63.0%) patients. A majority of patients were under 65 years old (*n* = 32, 69.6%) and most had a good ECOG performance status (PS) of 1–2 score (*n* = 41, 89.1%). Twelve patients (26.1%) had a smoking history and 12 patients (26.1%) had a family history of carcinoma. With regard to the metastatic site, the most common site was bone (*n* = 30, 65.2%) and next was brain (*n* = 14, 30.4%), liver (*n* = 10, 21.7%) in order. A total of 34 patients (73.9%) used a blood sample to detect *EGFR* T790M‐*cis*‐C797S mutation and 12 patients (26.1%) were detected by tissue sample, including lung tissue, pleural effusion, and ascites. The most common concomitant mutation was *EGFR* exon 19 deletion (*EGFR* 19del) (*n* = 29, 63.0%) followed by TP53 (*n* = 18, 39.1%), *EGFR* exon 21 p.L858R (*EGFR* 21L858R) (*n* = 8, 17.4%) and 6 patients were unknown (Table 1; Figure 1). Depending on the treatments, we divided patients into three groups and there were no obviously differences in baseline characteristics between the three groups (Table 1). In the univariant analysis, no baseline characteristics became a risk factor of PFS and for OS, ECOG PS 1–2 had a better survival outcome than ECOG PS 3–4 (11.73 vs. 2.73 months, hazard ratio [HR] = 0.25, 95% confidence interval [CI]: 0.09–0.74, *p* = 0.01). There was no significant difference in other baseline characteristics, including age, gender, smoking status, family history, central nervous system, and liver metastasis on PFS and OS (Table 1; Table S2).

**TABLE 1:** Baseline characteristics of patients

Baseline characteristics	Number of patients (*n*, %)	Brigatinib‐based therapy (*N* = 13)	Chemotherapy (*N* = 23)	Other targeted therapy (*N* = 10)^a^	*p*	Median PFS (months)	Univariant analysis		Multivariant analysis	
							95% CI	*p*	95% CI	*p*
Age(years)					0.09		0.45–1.75	0.73		
≤65	32 (69.6%)	10 (76.9%)	18 (78.3%)	4 (4.0%)		4.10				
>65	14 (30.4%)	3 (23.1%)	5 (21.7%)	6 (60.0%)		2.67				
Gender					0.21		0.56–1.96	0.88		
Male	17 (37.0%)	3 (23.1%)	8 (34.8%)	6 (60.0%)		4.10				
Female	29 (63.0%)	10 (76.9%)	15 (65.2%)	4 (40.0%)		3.40				
Smoking status					0.19		0.66–2.58	0.44		
Smoker	12 (26.1%)	2 (15.4%)	5 (21.7%)	5 (50.0%)		3.30				
Never‐smoker	34 (73.9%)	11 (84.6%)	18 (78.3%)	5 (50.0%)		4.20				
Family history					0.15			0.86		
Yes	12 (26.1%)	2 (15.4%)	6 (26.1%)	4 (40.0%)		2.37				
No	31 (67.4%)	11 (84.6%)	16 (69.6%)	4 (40.0%)		4.10				
Unknown	3 (6.5%)	0 (0.0%)	1 (4.3%)	2 (20.0%)		1.50				
ECOG PS					0.11		0.19–1.28	0.15	0.17–1.2	0.12
1–2	41 (89.1%)	12 (92.3%)	22 (95.7%)	7 (70.0%)		4.20			3	
3–4	5 (10.9%)	1 (7.7%)	1 (4.3%)	3 (30.0%)		1.33				
CNS metastasis					0.91		0.72–2.73	0.32		
Yes	14 (30.4%)	3 (23.1%)	8 (34.8%)	3 (30.0%)		3.23				
No	32 (69.6%)	10 (66.9%)	15 (65.2%)	7 (70.0%)		4.40				
Liver metastasis					0.46		0.26–1.23	0.15	0.27–1.90	0.50
Yes	10 (21.7%)	2 (15.4%)	7 (30.4%)	1 (10.0%)		4.57				
No	36 (78.3%)	11 (84.6%)	16 (69.6%)	9 (90.0%)		3.30				
C797S detection sample					0.75					
Blood	34 (73.9%)	9 (69.2%)	18 (78.3%)	7 (70.0%)						
Tissue	12 (26.1%)	4 (30.8%)	5 (21.7%)	3 (30.0%)						
Gene mutation					1.0		1.67–10.65	0.002	1.73–11.32	0.002
*EGFR* 21L858R	8 (17.4%)	3 (23.1%)	4 (23.5%)	1 (14.3%)		1.03				
*EGFR* 19del	29 (63.0%)	10 (76.9%)	13 (76.5%)	6 (85.7%)		4.57				
TP53	18 (39.1%)	6 (54.5%)	7 (31.8%)	5 (55.6%)	0.36	3.23	0.83–3.17	0.16		

Abbreviations: CI, confidence interval; CNS, central nervous system; ECOG, Eastern Cooperative Oncology Group; EGFR 19del, EGFR exon 19 deletion; EGFR 21L858R, EGFR exon 21 p.L858R; PFS, progression‐free survival; PS, performance status.

^a^ Other targeted therapy: like dacomtinib, bevacizumab, or a combined therapy of osimertinib and other targeted drugs.

### 3.2 Treatment and outcomes

**FIGURE 2:**
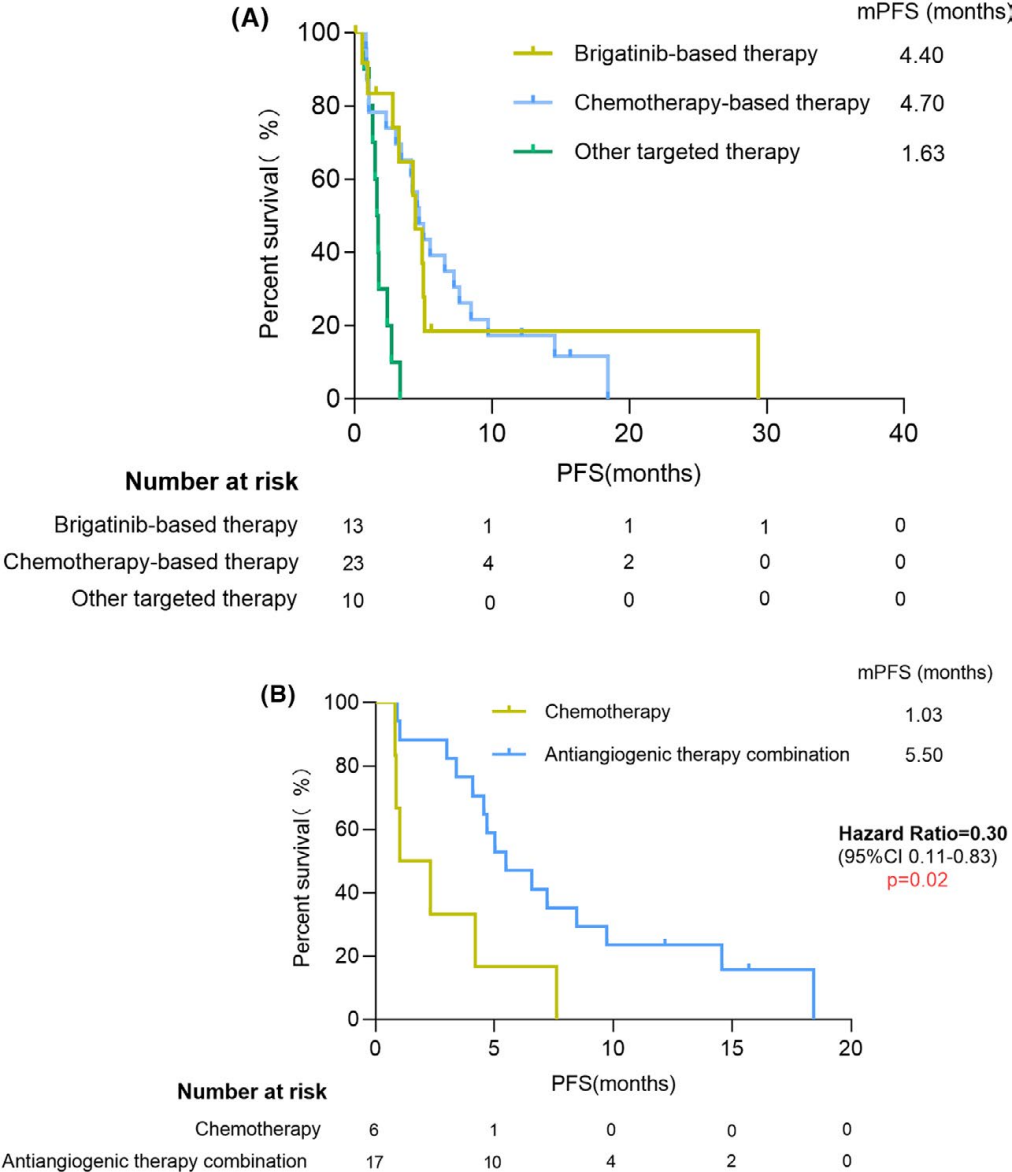
Treatment and progression‐free survival outcomes. (A) Kaplan–Meier curves in variant therapeutic strategies groups. (B) Kaplan–Meier curves of chemotherapy group and chemotherapy combined with antiangiogenic treatment

This study further analyzed all patients who have received therapy after *cis*‐C797S presence. A total of 23 patients received chemotherapy‐based therapy (50.0%), 13 patients (28.3%) applied brigatinib‐based treatment and 10 patients (21.7%) received other targeted treatments, like dacomtinib, bevacizumab, or a combined therapy of osimertinib, and other targeted drugs. The most common chemotherapy regimens were pemetrexed‐based regimens (*n* = 15). Seventeen patients tended to combine with anti‐angiogenics. The details of therapy regimens were illustrated in Tables S3 and S4. In the aspect of PFS, brigatinib‐based therapy (median PFS [mPFS]: 4.40 vs. 1.63 months, HR = 0.39, 95% CI: 0.21–0.73, *p* = 0.001) and chemotherapy‐based therapy (mPFS: 4.70 vs. 1.63 months, HR = 0.18, 95% CI: 0.06–0.50, *p* < 0.001) both exhibited a survival advantage compared to other targeted therapy (Figure 2A). However, for the OS, we have not discovered a similar tendency. The median OS of brigatinb‐based therapy, chemotherapy‐based therapy, and other targeted therapy were 9.93, 18.90, and 4.00 months, respectively. Although there was a numerical difference, the statistical significance did not reach between the three groups (Figure S1). In patients whose therapy efficacy could be evaluated, the DCR in patients who received brigatinib‐based therapy, chemotherapy‐based therapy, and other targeted therapy were 75.0% (9/12), 91.3% (21/23), and 30.0% (3/10), respectively (Table 2).

**TABLE 2:** Overall response to treatment as determined by RECIST v.1.1

Response	Brigatinib‐based therapy (*N* = 13)	Chemotherapy (*N* = 23)	Other targeted therapy (*N* = 10)
CR/PR	0	1	0
SD	9	20	3
PD	3	2	7
NA	1	0	0

Abbreviations: CR, complete response; NA, not available; PD, progressive disease; PR, partial response; RECIST, Response Evaluation Criteria in Solid Tumors; SD, stable disease.

We also conducted several subgroup analyses according to different variants. Considering the type of chemotherapy, the patients who underwent pemetrexed‐based treatment achieved a mPFS of 4.70 months, and in patients who received non‐pemetrexed treatment, the mPFS was 4.10 months (HR = 0.78, 95% CI: 0.31–1.99, *p* = 0.60). The median OS was 18.90 and 11.13 months in the two groups (HR = 0.56, 95% CI: 0.17–1.82, *p* = 0.33). Based on whether combining with anti‐angiogenic treatment, we divided the chemotherapy group into two subgroups. The patients who underwent anti‐angiogenic treatment had a superior PFS than patients who only received chemotherapy (mPFS: 5.50 vs. 1.03 months, HR = 0.30, 95% CI: 0.11–0.83, *p* = 0.02) (Figure 2B), however, a similar result has not been identified in OS (median OS: 18.90 vs. 22.80 months, HR = 1.73, 95% CI: 0.47–6.42, *p* = 0.41) (Figure S2). Furthermore, considering the comparable efficacy of brigatinib‐based treatment and chemotherapy and varied efficacy due to whether chemotherapy combines with anti‐angiogenic treatment, we compared the efficacy of brigatinib‐based therapy with chemotherapy alone. The median PFS of the two groups was 4.40 and 1.03 months (HR = 0.56, 95% CI: 0.20–1.59, *p* = 0.27).

### 3.3 Co‐mutation type and survival outcomes

**FIGURE 3:**
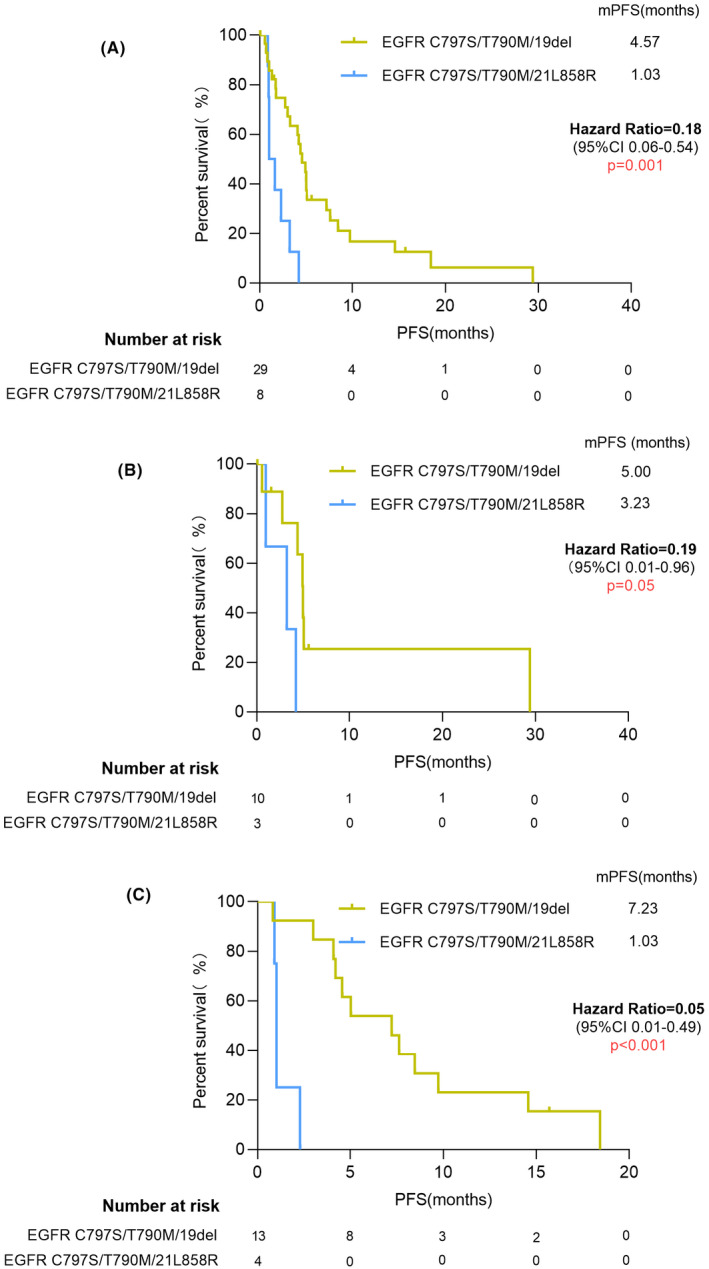
Kaplan–Meier curves of patients harboring *EGFR* C797S/T790M/19del and C797S/T790M/21L858R. (A) All patients. (B) Patients treated by brigatinib‐based therapy. (C) Patients treated by chemotherapy therapy

We analyzed the relationship between co‐mutation gene type and treatment efficacy. There were eight patients carrying *EGFR* 21L858R mutation and 29 patients carrying *EGFR* 19del mutation. In all patients carrying *EGFR* 19del mutation and *EGFR* 21L858R mutation, there was a significant difference on PFS of 4.57 and 1.03 months (HR = 0.18, 95% CI: 0.06–0.54, *p* = 0.001) (Figure 3A). No matter patients received brigatinib‐based therapy (5.00 vs. 3.23 months, HR = 0.19, 95% CI: 0.01–0.96, *p* = 0.05) (Figure 3B) or chemotherapy‐based treatment (7.23 vs. 1.03 months, HR = 0.05, 95% CI: 0.01–0.49, *p* < 0.001) (Figure 3C), the patients who carried *EGFR* 19del all presented a better treatment efficacy, especially in chemotherapy‐based treatment group. The was no significantly difference in OS of patients carrying *EGFR* 19del mutation and *EGFR* 21L858R mutation (11.13 vs. 5.63 months, HR = 0.95, 95% CI: 0.38–2.38, *p* = 0.91) (Figure S3). TP53 co‐mutation showed a poor prognosis in OS (5.90 vs. 18.90 months, HR = 2.45, 95% CI: 1.15–5.23, *p* = 0.02).

### 3.4 PDTO model and drug‐sensitivity test

**FIGURE 4:**
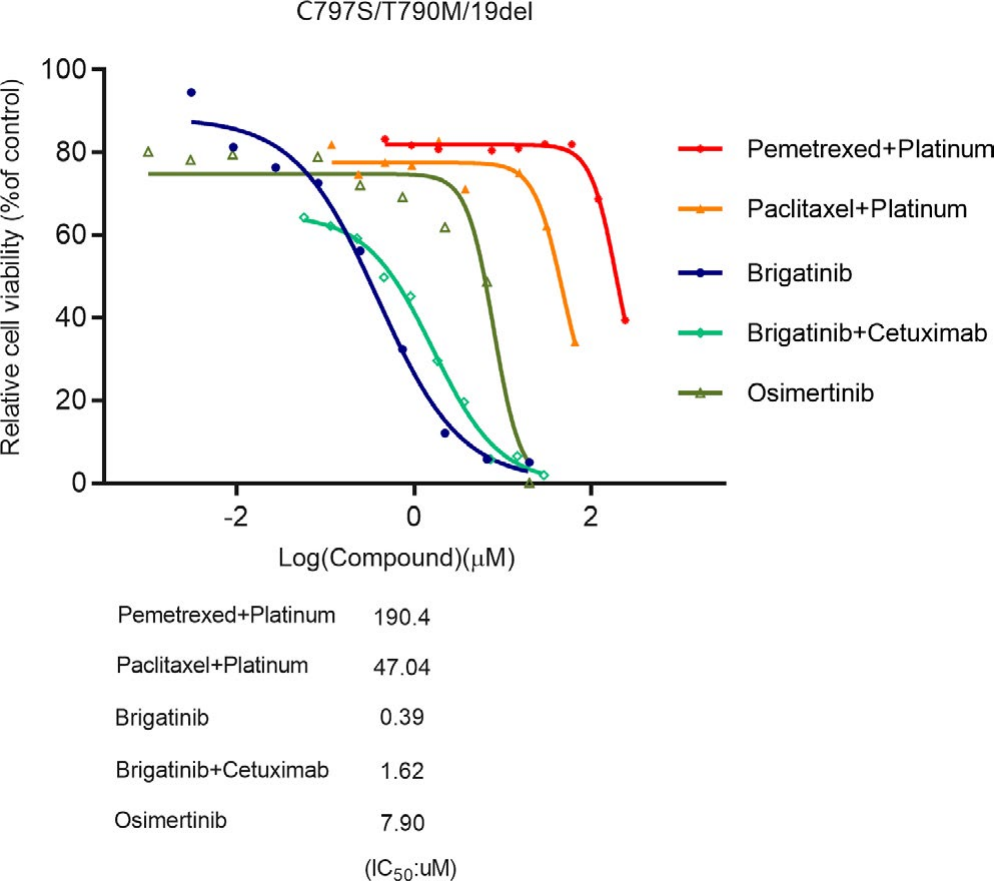
Drug sensitive test on patient‐derived tumor organoids (PDTO) model

The PDTO model technique is a cutting‐edge technology for in vitro three‐dimensional culture of tumor precision medicine.^14^, ^15^ The PDTO model can replicate the tissue complexity and genetic heterogeneity of tumors. In this model, we conducted a drug sensitivity test and brigatinib‐based therapy displayed a satisfied inhibitory activity. The brigatinib monotherapy, brigatinib combined with cetuximab, brigatinib combined with osimertinib achieved an inhibition rate of 81%, 88%, and 87%, respectively. However, the inhibition rate of chemotherapy, which was around 24%–30%, was unsatisfactory, and osimertinib had a 32% inhibition rate (Figure 4). Regretfully, due to the limitation of the technique, the test could not be conducted on chemotherapy combined with anti‐angiogenic therapy.

## 4 DISCUSSION

With the wide utilization of osimertinib due to the high percentage of *EGFR* mutation in the Asian population, many people inevitably develop resistance to osimertinib later. Albeit, Patients who acquired resistance to osimertinib were recommended to undergo initial systemic therapy options in the NCCN guideline and the clinical efficacy of these patients was still unknown. Previous studies have reported that the most common reason inducing resistance was *EGFR* C797S mutation and the incidence rate was around 7%–14%.^7^ In the cell lines which expressed C797S mutation, 85% of them presented the *cis*‐C797S and it showed non‐response to the *EGFR*‐TKIs.^8^ Our retrospective study provides real‐world evidence to investigate the clinical efficacy of various strategies for advanced NSCLC patients harboring *EGFR*‐T790M‐*cis*‐C797S. This study is also the largest analysis committed to metastatic lung adenocarcinoma patients with *cis*‐C797S mutation around the world to date.

Preclinical studies have detected the potent efficacy of cetuximab and brigatinib in C797S mutation cell lines.^9^, ^16^, ^17^ However, there were limited data to report the clinical efficacy in a real‐world setting so far. Two case reports elaborated on the clinical efficacy of brigatinib. In case one, the patient who received brigatinib combined with cetuximab had a desirable PFS of 9.00 months.^11^ And the other patient who underwent brigatinib, bevacizumab, and osimertinib reached the efficacy of PR and the treatment was still continuing.^12^ However, not all the patients who received brigatinib achieved a desirable efficacy. Another case report showed a patient who was treated by brigatinib and cetuximab progressed in only 1 month.^18^ A retrospective study compared the clinical efficacy of brigatinib combination with cetuximab and chemotherapy. In this study, a total of 15 patients were enrolled in which five patients received brigatinib combination with cetuximab and the PFS was 14 and 3 months.^13^ To sum up, there is still inconsistency about whether *cis*‐C797S patients could gain survival benefit by brigatinib and a lack of abundant evidence to illustrate the superiority of brigatinib to chemotherapy. In our study, the PFS of brigatinib‐based therapy, chemotherapy‐based therapy, and other targeted therapy were 4.40, 4.70, and 1.63 months, respectively. In addition, the PFS of chemotherapy combined with anti‐angiogenics and chemotherapy alone were 5.50 and 1.03 months, respectively. Bragatinib‐based therapy and chemotherapy combined with anti‐angiogenic treatment presented a possibility to become treatment options beyond the resistance of osimertinib. Although brigatinib (AP26113) was approved by Food and Drug Administration in 2017 for patients who were anaplastic lymphoma kinase positive, it has not been introduced in China mainland as yet. Therefore, if brigatinib was not available to some patients, considering the comparable efficacy, chemotherapy combined with anti‐angiogenic therapy could be an optional treatment.

This study also analyzed the relation of co‐mutation gene and treatment efficacy. The patients carrying *EGFR* 19del mutation had a longer PFS than patients carrying *EGFR* 21L858R mutation. Uchibori et al. have reported that brigatinib could inhibit *EGFR*‐triple mutation and C797S/T790M/L858R was less potent than in 19del.^9^ This conclusion was coincident with our real‐world study and may have a clinical indication of treatment options for various variant patients. Especially for patients who harboring the *EGFR* 19del, chemotherapy‐based therapy could bring a decent survival benefit as PFS reached 7.23 months.

Furthermore, we conducted a drug‐sensitive test on the patient‐derived tumor organoids model. This is a novel technology and can replicate the tissue complexity and genetic heterogeneity of the tumor. We cultured a model derived from one patient carrying *EGFR* C797S/T790M/19del mutation and found out that brigatinib and brigatinib combined with cetuximab showed a favorable inhibition rate of the model. However, the chemotherapy presented an unsatisfied efficacy which was in accordance with our real‐world study that the PFS of chemotherapy without anti‐angiogenic therapy was only 1.03 months which was much shorter than brigatinib‐based therapy. It is regrettable that as a result of the combination with anti‐angiogenic therapy cannot be conducted on PDTO model, we couldn't verify whether combination with anti‐angiogenic treatment had an equal efficacy of brigatinib‐based therapy.

Although our real‐world study precisely investigated the treatment strategies of *EGFR* T790M‐*cis*‐C797S mutated patients, as well as provided a detailed description of clinical characteristics, several limitations cannot be ignored. First, this was a retrospective study that easily appeared selection bias. Second, the heterogeneity of patients in this real‐world study in terms of specific treatment regimens was considered a limitation. Third, the number of patients with *EGFR* 21L858R was limited, which led to preventing us from providing corroborative information on the clinical efficacy of brigatinib. Last but not least, the PDTO model was derived from one patient and an anti‐angiogenic regimens could not be tested on this model which might lead to the bias of drug‐sensitive test. Although our sample size was the largest around the world, more clinical evidence as well as cell lines and patient‐derived xenograft model are still urgently needed to correspond to our findings and further draw a clear conclusion. We believe that our study provides valuable evidence to give suggestions on clinical therapy in Chinese *cis*‐C797S‐mutated NSCLC patients to a certain extent.

## 5 CONCLUSION

In this retrospective study, clinical characteristics and real‐world clinical practice in advanced NSCLC patients harboring *EGFR* T790M‐*cis*‐C797S mutation were observed. Brigatinib‐based therapy and chemotherapy combined with anti‐angiogenic therapy are considerable after progression beyond osimertinib treatment. Patients carried *EGFR* 19del/T790M/*cis*‐C797S mutation achieved a better survival benefit than patients harboring *EGFR* 21L858R/T790M/*cis*‐C797S mutation.

## CONFLICT OF INTEREST

The authors declare that they have no conflict of interest.

## Supporting information

Fig S1

Fig S2

Fig S3

Table S1‐S4

## Data Availability

The datasets generated during and/or analyzed during the current study are available from the corresponding author on reasonable request.
