# Supplementary figures and images for: Possibility of brigatinib‐based therapy, or chemotherapy plus anti‐angiogenic treatment after resistance of osimertinib harboring EGFR T790M‐cis‐C797S mutations in lung adenocarcinoma patients
Source: Cancer Med. 2021 Oct 6;10(23):8328–37. doi: 10.1002/cam4.4336 (PMC8633234; doi:10.1002/cam4.4336)

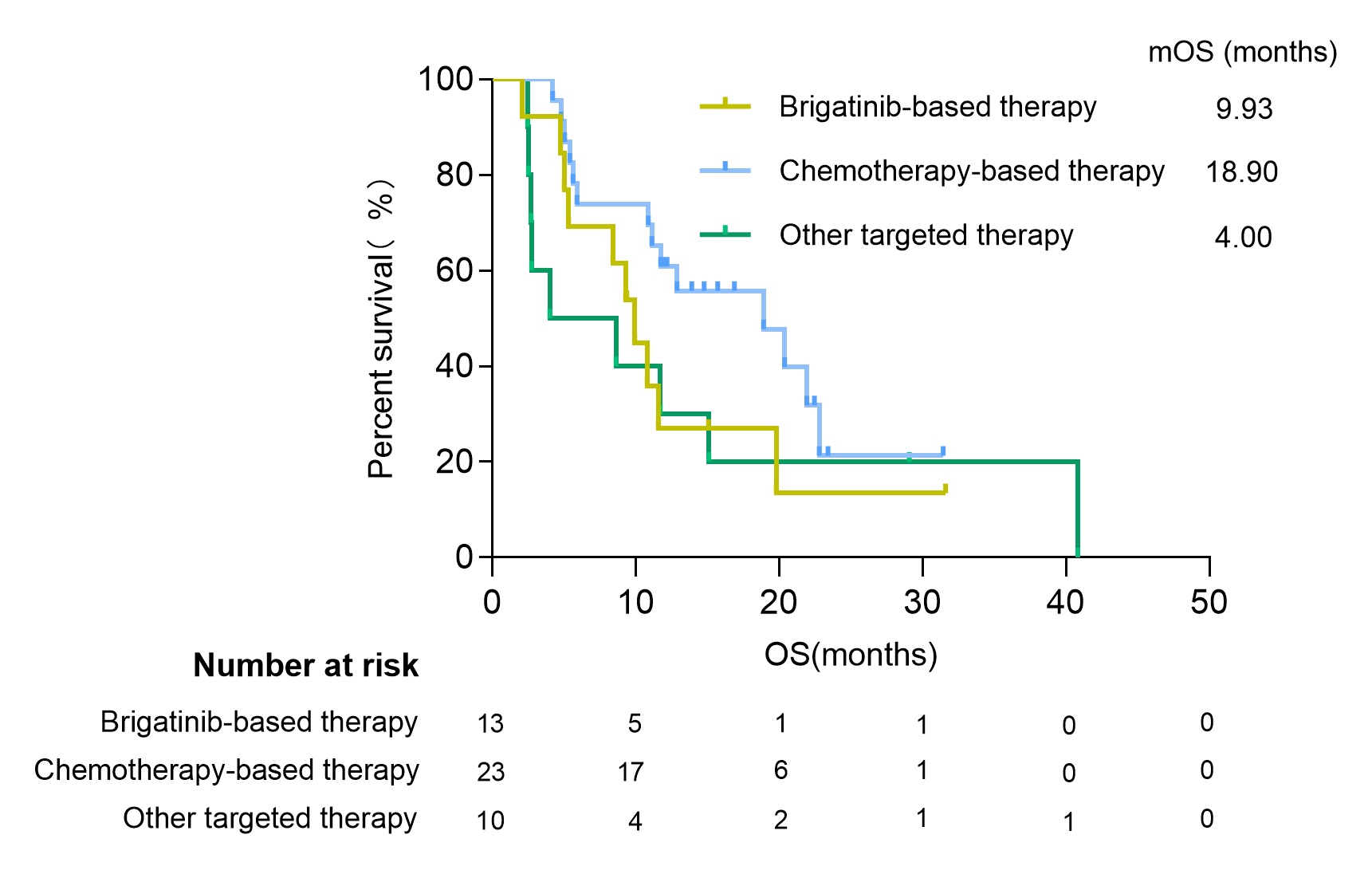

Supplement: Supplementary file 1 — Fig S1 [file CAM4-10-8328-s002.tif]

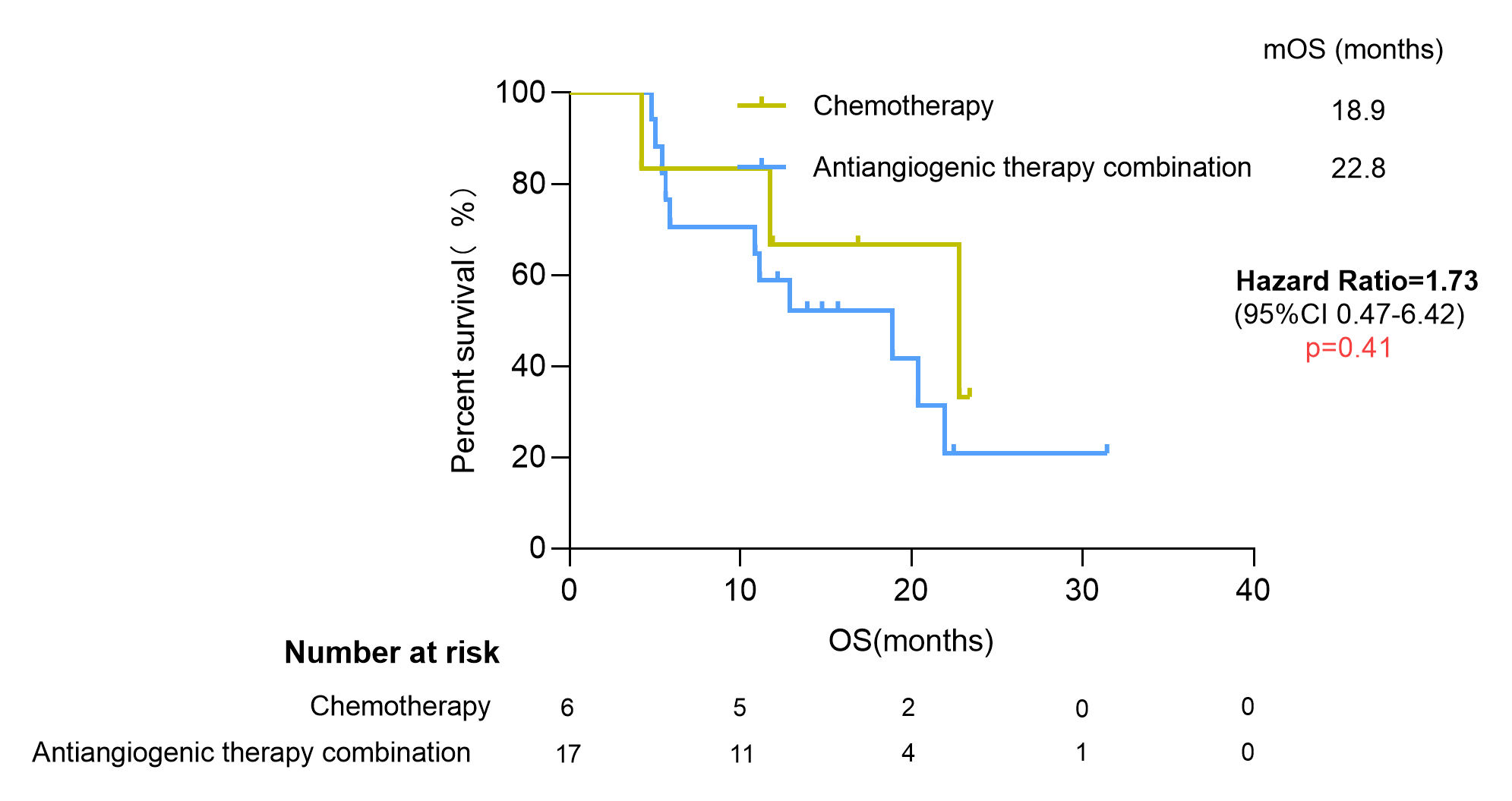

Supplement: Supplementary file 2 — Fig S2 [file CAM4-10-8328-s001.tif]

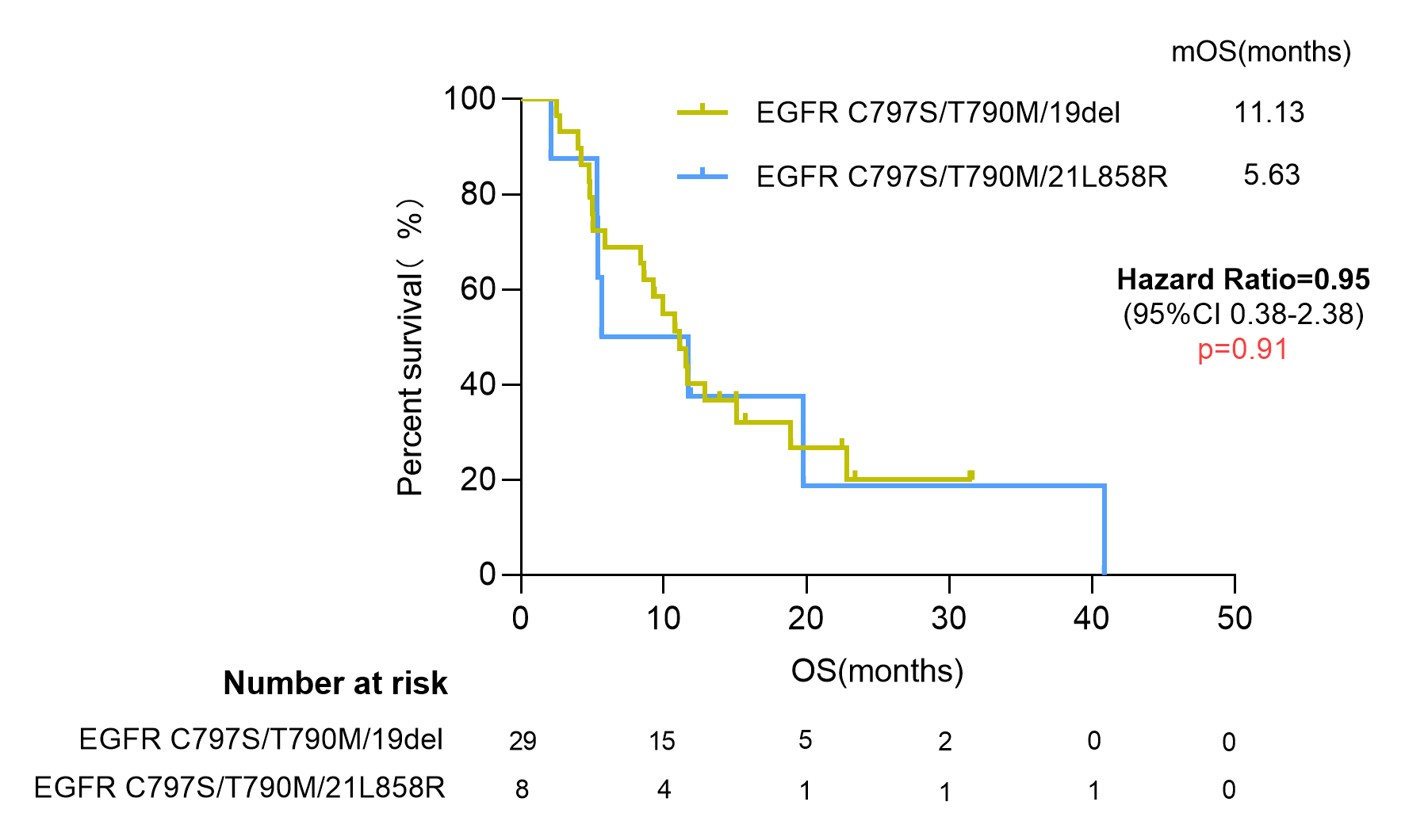

Supplement: Supplementary file 3 — Fig S3 [file CAM4-10-8328-s004.tif]
