# Supplementary material for: Possibility of brigatinib‐based therapy, or chemotherapy plus anti‐angiogenic treatment after resistance of osimertinib harboring EGFR T790M‐cis‐C797S mutations in lung adenocarcinoma patients
Source: Cancer Med. 2021 Oct 6;10(23):8328–37. doi: 10.1002/cam4.4336 (PMC8633234; doi:10.1002/cam4.4336)
Supplement: Supplementary file 4 — Table S1‐S4 [file CAM4-10-8328-s003.docx]

**Supporting Information**

**Article title:** Possibility of brigatinib-based therapy, or chemotherapy plus anti-angiogenic treatment after resistance of osimertinib harboring EGFR T790M-cis-C797S mutations in lung adenocarcinoma patients

**Author names:** Yaning Yang^1*^, Haiyan Xu^2*^, Li Ma^3*^, Lu Yang^1^, Guangjian Yang^1^, Shuyang Zhang^1^, Xin Ai^1^, Shucai Zhang^3^, Yan Wang^1^

**Author affiliation:**

^1^ Department of Medical Oncology, National Cancer Center/National Clinical Research Center for Cancer/Cancer Hospital, Chinese Academy of Medical Sciences and Peking Union Medical College, Beijing, China.

^2^ Department of Comprehensive Oncology, National Cancer Center/National Clinical Research Center for Cancer/Cancer Hospital, Chinese Academy of Medical Sciences and Peking Union Medical College, Beijing, China.

^3^ Department of Medical Oncology, Beijing Tuberculosis and Thoracic Tumor Research Institute, Beijing Chest Hospital, Capital Medical University, Beijing, China.

*These authors contributed equally to this work.

**E-mail address of the corresponding author:**

Yan Wang: [wangyanyifu@163.com](mailto:wangyanyifu@163.com)

Shucai Zhang: [sczhang6304@163.com](mailto:sczhang6304@163.com)

**Supplementary Table 1 Flow Chart**

13 patients were excluded

Stage I/II(n=2)

No measurable lesions(n=2)

No any treatment (n=9)

10 patients received other targeted therapy

23 patients received chemotherapy

13 patients received brigatinib-based therapy

**Supplementary Table 2 Univariant analysis of overall survival**

| Variants | Median OS (m) | Univariant analysis | | |
| --- | --- | --- | --- | --- |
|  |  | **HR** | **95%CI** | **P** |
| Age(years)  ≤65  >65 | 11.57  9.3 | 0.92 | 0.43-1.99 | 0.83 |
| Gender  Male  Female | 11.57  11.73 | 1.09 | 0.53-2.21 | 0.82 |
| Smoking status  Smoker  Never-smoker | 8.63  11.73 | 1.52 | 0.72-3.21 | 0.28 |
| Family history  Yes  No  Unknown | 10.80  12.87  11.13 |  |  | 0.52 |
| ECOG  1-2  3-4 | 11.73  2.73 | 0.25 | 0.09-0.74 | 0.01 |
| CNS metastasis  Yes  No | 5.33  11.73 | 1.48 | 0.70-3.15 | 0.30 |
| Liver metastasis  Yes  No | 11.73  11.13 | 0.97 | 0.42-2.25 | 0.95 |
| Gene mutation  EGFR 21L858R  EGFR 19del | 5.63  11.13 | 1.05 | 0.42-2.61 | 0.91 |
| TP53 mutation  Yes  No | 5.90  18.90 | 2.45 | 1.15-5.23 | 0.02 |

**Abbreviations**: OS: overall survival; ECOG: Eastern Cooperative Oncology Group; CNS: central nervous system; EGFR 21L858R: EGFR exon 21 p.L858R; EGFR 19del: EGFR exon 19 deletion.

**Supplementary Table 3 The details about therapy**

| Therapy | Number of patients (n%) |
| --- | --- |
| Total | 46 |
| *Chemotherapy*  Regimen with Pemetrexed  Pemetrexed/Platinum/Bevacizumab  Pemetrexed/Bevacizumab  Pemetrexed/Platinum  Pemetrexed | 23 (50.0)  15 (32.6)  9 (19.6)  2 (4.3)  3 (6.5)  1 (2.2) |
| Regimen without Pemetrexed  Paclitaxel/Platinum/Bevacizumab  Paclitaxel/Navelbine/Bevacizumab  Gemcitabine/Platinum/Endostar  Docetaxel/Bevacizumab  Paclitaxel/Bevacizumab  Paclitaxel/Platinum  Paclitaxel | 8 (17.4)  2 (4.3)  1 (2.2)  1 (2.2)  1 (2.2)  1 (2.2)  1 (2.2)  1 (2.2) |
| *Brigatinib-based therapy*  Brigatinib/Cetuximab  Brigatinib/Osimertinib  Brigatinib | 13 (28.3)  10 (21.7)  2 (4.3)  1 (2.2) |
| *Other targeted therapy*  Osimertinib/Gefitinib  Osimertinib/Bevacizumab  Osimertinib/Apatinib  Osimertinib  Dacomtinib  Bevacizumab | 10 (21.7)  2 (4.3)  1 (2.2)  1 (2.2)  4 (8.7)  1 (2.2)  1 (2.2) |

**Supplementary Table 4 Clinical Details of patients who received brigatinib-based therapy**

| Patient Number | Gender | Age (year) | Brain Metastasis | Liver  Metastasis | Treatment Received | Best Response | PFS (month)^a^ | Adverse  Events |
| --- | --- | --- | --- | --- | --- | --- | --- | --- |
| P1 | Female | 64 | Yes | No | Brigatinib+cetuximab | SD | 3.23 | Paronychia (G1); Rash (G1) |
| P2 | Female | 51 | No | No | Brigatinib+cetuximab | SD | 5.10 | - |
| P3 | Female | 53 | Yes | Yes | Brigatinib+cetuximab | SD | 29.40 | Weakness (G1); Dizzy (G1) |
| P4 | Female | 66 | No | No | Brigatinib+cetuximab | SD | 4.40 | - |
| P5 | Female | 67 | No | No | Brigatinib | N/A | 0.07 | Interstitial pneumonia (G4) |
| P6 | Male | 50 | No | No | Brigatinib+cetuximab | PD | 0.57 | Tachycardia (G2) |
| P7 | Male | 39 | No | No | Brigatinib+cetuximab | SD | 4.23 | Rash (G4) |
| P8 | Female | 49 | No | No | Brigatinib+cetuximab | SD | 1.57+ | - |
| P9 | Female | 56 | No | Yes | Brigatinib+osimertinib | SD | 2.77 | - |
| P10 | Female | 35 | No | No | Brigatinib+cetuximab | PD | 5.00 | - |
| P11 | Female | 60 | Yes | No | Brigatinib+cetuximab | SD | 4.93 | - |
| P12 | Male | 72 | No | No | Brigatinib+osimertinib | SD | 5.60 | - |
| P13 | Female | 63 | No | No | Brigatinib+cetuximab | PD | 0.97 | - |

^a^PFS time marker with + indicates the patients were still receiving the treatment until the last follow-up time, August 1st, 2021.

G1: grade 1; G2: grade 2; G3: grade 3; G4: grade 4; SD: stable disease; PD: progressive disease; PFS: progression-free survival

**Supplementary Figure legends**

**Supplementary Figure 1** Kaplan-Meier curves of overall survival (OS) in various kinds of treatments

**Supplementary Figure 2** Kaplan-Meier curves of OS in patients treated with chemotherapy and anti-angiogenics or not

**Supplementary Figure 3** Kaplan-Meier curves of OS in patients harboring EGFR C797S/T790M/19del and C797S/T790M/21L858R
